# Phages of *Listeria* offer novel tools for diagnostics and biocontrol

**DOI:** 10.3389/fmicb.2014.00159

**Published:** 2014-04-10

**Authors:** Steven Hagens, Martin J. Loessner

**Affiliations:** ^1^Micreos Food SafetyWageningen, Netherlands; ^2^Institute of Food, Nutrition and Health, ETH ZürichZürich, Switzerland

**Keywords:** bacteriophage, tools, diagnostics, typing, biocontrol, endolysins

## Abstract

Historically, bacteriophages infecting their hosts have perhaps been best known and even notorious for being a nuisance in dairy-fermentation processes. However, with the rapid progress in molecular microbiology and microbial ecology, a new dawn has risen for phages. This review will provide an overview on possible uses and applications of *Listeria* phages, including phage-typing, reporter phage for bacterial diagnostics, and use of phage as biocontrol agents for food safety. The use of phage-encoded enzymes such as endolysins for the detection and as antimicrobial agent will also be addressed. Desirable properties of candidate phages for biocontrol will be discussed. While emphasizing the enormous future potential for applications, we will also consider some of the intrinsic limitations dictated by both phage and bacterial ecology.

## INTRODUCTION

Bacteriophages affect the world in numerous ways. They represent parasites of bacteria, without own metabolism, relying on the host cell to provide energy and resources for their own replication. In terms of quantity, phages are the most abundant self-replicating entities on our planet, and outnumber their hosts by a factor of at least 10:1. This results in an estimated population of 10^30^–10^31^ phage particles existing on the planet at any given time (Wommack and Colwell, 2000). In order to maintain this number, an estimated 10^25^ infections happen every second (Pedulla et al., 2003). In any environment where bacteria play an important role it therefore stands to reason that bacteriophage will have an impact on that environment. In the worlds’ oceans and seas cyanobacteria play a vital role in primary production and most work focusing on ecological impact of phages has focused on their role in nutrient cycles. However, it should be obvious that, next to protozoa, phages are the only natural predators of bacteria and thus play an essential role in controlling bacterial populations everywhere. Bacteriophages also influence human lives in various other ways. Wherever bacteria are used in fermentation processes, be it for food or production of other compounds, phages can affect the end-result by infecting and lysing the desired bacteria. In the biological sciences, bacteriophages have been instrumental in building our understanding of fundamental genetic principles, such as the concept of the operon, functioning of promoters and the restriction-modification, one of the most valuable and versatile tools in molecular biology. Phages can contribute to the virulence of bacterial pathogens in two ways. General transduction is a process where instead of phage-DNA host-DNA is packaged into infectious phage-like particles and this host DNA is subsequently injected into a susceptible host where it may integrate into the chromosome through homologous recombination. Whenever this transduced DNA contains virulence genes the infected bacteria can have altered virulence. Although other strategies for survival and replication exist, most phages can be separated into temperate or virulent phages. The latter are often termed lytic or strictly lytic as well. This lifestyle involves obligate production of new phages after infection resulting in lysis of the host and release of progeny phage. Temperate phages can do this but alternatively they can integrate their genetic information into the host chromosome. Lysogenic conversion is the phenomenon where temperate phages alter the virulence of the infected host after DNA integration. Examples of bacteria that rely on phage (genes) for their full pathogenic potential are *Vibrio cholera*, which is only pathogenic when it the cholera toxin is introduced by phage CTXϕ, the diphtheria toxin of *Corynebacterium diphteriae* is also encoded by a phage, and shiga-toxin production in Enterobactericeae originates from genes on temperate phages integrated into the genomes of these bacteria (Freeman, 1951; Smith et al., 1983; Waldor and Mekalanos, 1996). Many more examples can be found in the literature. Lastly, phages have long been used in medical applications. Phage-typing has long been employed to conduct epidemiological studies of bacterial infection.

Over the past two decades, there has been a huge revival of interest in studying phages, but for other reasons. The emergence and increase in antibiotic-resistant bacteria and the possibility of replacing chemical drugs with phage or phage-encoded enzymes has been one of the main driving forces behind this renaissance. The fundamental insights which the genomics era has provided us with also led to the development of various tools based on phages or their components. Phage protein based detection of bacteria is now commercially available in the form of Vidas UP^®^ technology, which employs recombinant host recognition proteins for *in vitro* diagnostics (IVD) of *Escherichia coli* and *Salmonella*. Phages have also been considered and applied since many decades to treat bacterial infections. This practice was and still is most common in the former Soviet Union, and to this day, phage preparations to treat a variety of infections are still produced in the Republic of Georgia. In the Western world, phage products for biocontrol are currently being commercialized for agricultural applications and food applications. Phage-derived proteins have also been commercialized and a number of commercial companies are investigating phage or phage-derived products for use in medical applications. The following chapters will focus on the impact of phage studies on our understanding of the genus *Listeria* with emphasis on *Listeria monocytogenes* as well as phage-derived tools for the detection and biocontrol of this important foodborne pathogen.

## THE GENUS *Listeria*

*Listeria* spp. are small Gram-positive, flagellated rods with a ubiquitous distribution in the environment. Their principle lifestyle is saprophytic and thus the bacteria are frequently associated with plant material. Nine species are currently recognized, and they are *L. monocytogens*, *L. ivanovii, L. innocua, L. marthii, L. rocourtiae, L. seeligeri, L. welshimeri L. fleischmannii,* and *L. grayii.* Most research has been undertaken with respect to the human pathogen *L. monocytogenes* (Glaser et al., 2001; Graves et al., 2010; Leclercq et al., 2010; Bertsch et al., 2013). In healthy individuals, the infection (termed listeriosis) often manifests itself as a gastroenteritis, but in the very young, old, pregnant, and immunocompromised (a risk group commonly referred to as YOPIs), the bacteria can penetrate the intestinal tract and cause systemic infections including the central nervous system, and stillbirth or abortion in pregnant women (Vázquez-Boland et al., 2001). The disease is caused by the consumption of food contaminated with *L. monocytogenes*. The bacterium is a psychrotroph and can grow at refrigeration temperatures, and tolerates a wide range of pH and salt concentrations, rendering even low contaminations at production level dangerous if the shelf life of the food is longer than a few days (Gandhi and Chikindas, 2007). Many countries have strict regulations on the number of *L. monocytogenes* cells that are allowed in a given food, and zero-tolerance policies are in place in the US and Oceania. Phage therapy of Listeriosis is of course no option, as the bacteria multiply as intracellular pathogens, and cannot be reached by phages. Even antibiotics to which the bacteria are usually highly sensitive need to be administered at relatively high doses over prolonged periods of time in order to establish sufficient intracellular concentration. However, phage can be used both for the detection of this pathogen, and in controlling their presence in food processing equipment and on ready-to-eat products.

## BACTERIOPHAGES OF *Listeria*

*Listeria* phages have been isolated from several of sources, including sewage plants, silage, food processing environments, and from lysogenic strains (Loessner and Busse, 1990; Hodgson, 2000; Kim et al., 2008; Arachchi et al., 2013). In total, some 500 phages specifically infecting *Listeria* have been identified, while only a limited number has been fully characterized on the molecular and genomic level (Loessner et al., 2000; Zimmer et al., 2003; Carlton et al., 2005; Dorscht et al., 2009; Schmuki et al., 2012). Phages have been reported to be able to infect other species. These studies revealed extensive mosaicism between many *Listeria* phages. All known *Listeria* phages belong to the order *Caudovirales*, which are tailed viruses with double stranded DNA. Podoviruses feature very short tails, while myoviruses have long, non-flexible contractile tail and siphoviruses have long, flexible tails. While members of both the *Siphoviridae* and *Myoviridae* are common, no *Podoviridae* infecting *Listeria* have yet been found and reported. The majority of the described phage are temperate, being able to integrate into the host genome. In fact, many *Listeria* strains are lysogenic, sometimes carrying multiple prophages, while most also harbor defective prophages. Many *Listeria* strains produce and release so-called monocins, which represent defective prophages, often recognized as lytic particles resembling phage tail structures (Zink et al., 1994, 1995). As in other bacteria, exposure to DNA-damaging agents such as UV light or Mitomycin C can induce the lytic cycle and lead to production of infective phage (Loessner, 1991). *Listeria* temperate phages generally display narrow host ranges, infecting only a small percentage of strains. This is in part caused by homoimmunity, i.e., resistance to a particular phage based on presence of a compatible repressor protein encoded by the same or a related phage within the bacterial cell. It is also noteworthy that the temperate phages of *Listeria* appear to be largely serovar-specific. It has been shown that this is based upon cell wall ligands, which the temperate phages recognize and attach to infect their hosts. They are serovar-specific sugar substituents of the polyribitol-phosphate teichoic acids on the *Listeria* cell wall (Wendlinger et al., 1996). In contrast, the large virulent phages such as A511 have been shown to use the cell-wall peptidoglycan backbone itself as a primary receptor (Wendlinger et al., 1996; Habann et al., 2014). Phages specifically infecting the different major serovar groups (1/2, 4, 5, and 6) have been identified, while no phages for *L. monocytogenes* serovar 3 or *L. grayii* strains have yet been found. Very little data exist on phenotype conversion or alteration following lysogenization. A recent study shows that prophage integration into the *Listeria comK* gene hinders cells escaping from macrophage phagosomal environments. In this case, the prophage may be excised during intracellular growth without generating progeny phage, integrity of *comK* is reconstituted, and following a cascade of events triggered by the ComK protein, the bacteria can eventually escape (Rabinovich et al., 2012). Another study shows that cells which harbor a phage integrated into *comK* display enhanced persistence and higher cell densities under meat processing plant conditions compared to strains lacking the phages. However, no explanation is offered and the underlying mechanisms causing this phenomenon remain obscure (Verghese et al., 2011). The less frequent virulent phages of *Listeria* fall into three groups. The large (~140 kb) Myoviruses P100 and A511 (Carlton et al., 2005; Klumpp et al., 2008) feature exceptionally broad host ranges, while the smaller siphoviruses P35 and P40 (around 40 kb) and the recently described, slightly larger (70 kb) phage designated P70 (Dorscht et al., 2009; Schmuki et al., 2012) still infect most members of given serovar groups. Virulent phages, i.e., those that will always kill their host cells are of course most useful when phage-based biocontrol is the aim. In contrast, generalized transducing phages, even though they may feature a virulent lifestyle such as P35 (Hodgson, 2000), are not desirable for application in biocontrol because they may tranfer genetic material, including virulence factors from one strain to another (Hagens and Loessner, 2010). Preferred are members of a specific group of the large, virulent myoviruses closely related to P100 and A511, which have been isolated from sources in Europe, the US and New Zealand (Zink and Loessner, 1992; Carlton et al., 2005; Kim et al., 2008; Bigot et al., 2011; Arachchi et al., 2013).

The natural property of phage to specifically target and kill bacterial cells of a certain species or genus has been fairly well exploited with respect to *Listeria*. As mentioned above, phage therapy of *L. monocytogenes* infections is not possible. However, prevention of the disease by anti-*Listeria* control measures during and after food processing is highly effective and desirable. Phages used for biocontrol purposes should be virulent and feature a broad host range, i.e., infect and kill as many target strains as possible. They must also be unable to transduce, and should ideally be propagated on a non-pathogenic production strain, or alternatively needs to be purified from the bacterial lysate. As of now, two *Listeria* phage products are commercially available: a broad host range myovirus is marketed as “Listex^TM^ P100,” and was granted GRAS (Generally Recognized As Safe) status by FDA and USDA in 2007 for use in all food products. Several studies have demonstrated its efficacy in foods such as cheese, RTE (ready to eat) meats and poultry, fruits, vegetables, and smoked fish, either alone or in combination with a growth limiting antimicrobial (Carlton et al., 2005; Soni and Nannapaneni, 2010a; Soni et al., 2010, 2012; Chibeu et al., 2013; Oliveira et al., 2014). Another study showed its efficacy on stainless steel surfaces, achieving more than five logs bacterial kill (Soni and Nannapaneni, 2010b). Another study used phage A511, closely related to P100, and confirmed its effectiveness in various RTE foods and surface ripenend soft cheeses (Guenther et al., 2009; Guenther and Loessner, 2011). Another product is termed “ListShield,” and is also FDA and USDA approved. It consist of a cocktail of several phages, and its efficacy was investigated in two studies using fresh-cut produce in combination with the bacteriocin Nisin and as a spray application on honey-dew melons (Leverentz et al., 2003, 2004). An alternative approach to suspended phage is directed immobilization of the viral particles on cellulose membranes, which has been proposed as a possible intervention strategy against *Listeria* on packaging material (Anany et al., 2011).

## TOOLS BASED ON *Listeria* PHAGE

**Table 1** lists several *Listeria* phage-derived applications. The first widely used application was in phage typing. This is an inexpensive, low-tech and relatively quick tool to differentiate strains in epidemiological and microbial source-tracking studies. While being increasingly replaced by modern and more sophisticated molecular methods, it is yet not likely to become completely obsolete because of its simplicity and low cost. Phage typing had a prominent role in demonstrating food to be the primary cause of listeriosis in humans (Fleming et al., 1985). A number of different phage sets were developed (Audurier et al., 1979, 1984; Rocourt et al., 1985; McLauchlin et al., 1986; Loessner and Busse, 1990; Loessner, 1991; Estela and Sofos, 1993; Gerner-Smidt et al., 1993; van der Mee-Marquet et al., 1997). However, since *Listeria* serovar 3 strains appear resistant to phage infection, not all isolates can be typed. However, almost all strains that occur in foodborne outbreaks feature either serovar 1/2 or 4b, and are mostly susceptible to phage infection and can therefore generally be differentiated using phage typing (van der Mee-Marquet et al., 1997). Transducing phages, while not suited for biocontrol purposes, have been used to transfer genetic traits from one strain to another, allowing genetic manipulation and phenotype investigations (Freitag, 2000; Hodgson, 2000). Lauer and coworkers provided specific *E. coli*/*Listeria* shuttle vectors designed to integrate into the *Listeria* chromosome after transformation. Here, site-specific integration by *Listeria* phage integrase has been employed to mediate single-copy, selection-free integration of genes placed on the vectors. As a proof of concept, the approach allowed successful complementation of *actA* and *hly* knockouts (Lauer et al., 2002). Derivatives of the initial pPL2 vector have since been used in many studies addressing *L. monocytogenes* phenotypes and virulence properties, have been successfully employed to monitor promoter activity in *in vivo* infection studies (Lenz and Portnoy, 2002; Lenz et al., 2003; Grundling et al., 2004; Bron et al., 2006), and were fundamental in elucidating the mechanisms of L-form formation in *L. monocytogenes* (Dell’Era et al., 2009).

**Table 1:** *Listeria* phage-derived tools.

Phage or phage components	Application	Reference
Large sets of different phages (broad and narrow host range)	Phage typing	Audurier et al. (1979,1984), Rocourt et al. (1985), McLauchlin et al. (1986), Loessner and Busse (1990), Loessner (1991), Estela and Sofos (1993), Gerner-Smidt et al. (1993), van der Mee-Marquet et al. (1997)
Single phages or cocktails (broad host range)	Biocontrol (targeted killing in foods)	Leverentz et al. (2003,2004), Carlton et al. (2005), Guenther and Loessner (2011), Guenther et al. (2009), Soni et al. (2010,2012), Soni and Nannapaneni (2010a), Chibeu et al. (2013),Oliveira et al. (2014)
Reporter phages (broad host range)	Diagnostics (detection)	Loessner et al. (1996,1997), Hagens et al. (2011)
Phage endolysins and functional domains	Diagnostics	Kretzer et al. (2007), Schmelcher et al. (2010), Walcher et al. (2010), Tolba et al. (2012)
Phage tail fibers	Immobilization and detection	Junillon et al. (2012), Habann et al. (2014)
Endolysins and modified endolysins	Antimicrobial	Fischetti (2010), Schmelcher et al. (2012)
Phage integrase genes	Site-specific integration vectors	Lauer et al. (2002)

Rapid and efficient detection of a *Listeria* contamination is critical with respect to food safety, and for compliance of food production with regulations concerning the presence of *L. monocytogenes* in foods and on food-contact surfaces. The high specificity of phages combined with the fact that viruses only multiply in viable and active host cells has sparked the development of phage amplification-based assays for the detection of various pathogens, including *Listeria*. One possible use is measuring an increase of *Listeria* phages after incubation with a contaminated sample (Bakulov et al., 1984). However, genetically modified reporter phages carrying a gene encoding an easy-to-measure product or activity offer more interesting and versatile alternatives. The reporter gene product will only be made following successful phage infection and gene expression. *Listeria* reporter phage A511::*luxAB* transduces a *luxAB* gene fusion coding for bacterial luciferase (Loessner et al., 1996), and its efficacy in rapid detection of low-level *Listeria* contaminations from various foods has been demonstrated (Loessner et al., 1997). The same phage was used to construct A511::*celB,* featuring a thermophilic glycosidase of *Pyrococcus furiosus*. Reporter genes from thermophiles feature an exceptional ease-of-use, and allow a wide range of substrates and assay formats to choose from (Hagens et al., 2011).

Rather than employing complete phages as antimicrobials and for diagnostics, proteins and peptides from phage may also be used for this purpose. Phage-encoded cell-wall-hydrolyzing enzymes are particularly useful. In order to digest the bacterial cell wall prior to release of phage progeny, most phages produce a class of enzymes termed endolysins. These enzymes feature a two-domain structure with an N-terminal catalytic domain and a C-terminal cell wall-binding domain (CBD) that binds with high affinity to cell wall-associated ligands, in order to direct the catalytic activity to its target site, and at the same time prevent collateral damage and lysis of yet uninfected potential host cells (Loessner et al., 1995). Such endolysin peptidoglycan hydrolases have a huge potential as antimicrobial reagents against Gram-positive pathogens, which has been reviewed elsewhere (Fischetti, 2010; Schmelcher et al., 2012). Yet, application of *Listeria* phage endolysins as highly specific antimicrobial agent in foods is hampered by the specific requirements of these enzymes, in terms of pH and salt concentration. Endolysins from *Listeria* phages can, however, be useful for removing *Listeria* occurring on food-contact surfaces and in biofilms.

Due to the high binding affinity and specificity of the endolysin CBD, these protein domains have been explored and used as versatile tools for diagnostics and identification of *Listeria*. Studies have shown that CBDs immobilized on paramagnetic beads are very effective in order to separate target cells from dilute suspensions, and the technique can be elegantly combined with different secondary diagnostic steps (Kretzer et al., 2007; Walcher et al., 2010). A recent study investigated detection of *Listeria* cells using electrochemical impedance spectroscopy (EIS), which can measure bacteria captured by the CBD molecules immobilized on a gold screen printed electrode (SPE; Tolba et al., 2012). Differently colored fluorescent proteins fused to CBDs with different recognition and binding spectra allow for rapid and multiplexed detection and differentiation of *Listeria* strains by fluorescence microscopy (Schmelcher et al., 2010). As an alternative approach, *Listeria* phage tail fiber proteins coupled to a solid support can also be used to bind target cells for primary enrichment and subsequent detection (Junillon et al., 2012).

In summary, the past years have demonstrated that phages infecting *Listeria* and other bacteria (Hagens and Loessner, 2010) provide us with numerous novel tools for various interesting approaches. The evolution of molecular microbiology and genetic engineering as technology platform, together with insights into phage biology, have been combined in interesting ways, and it is likely that this process will continue and more tools harnessing the properties and incredible biological specificity of bacterial viruses will be developed and available in the future.

## Conflict of Interest Statement

The authors declare that the research was conducted in the absence of any commercial or financial relationships that could be construed as a potential conflict of interest.

## References

[B1] Anany H. Chen W. Pelton R. Griffiths M. W. (2011). Biocontrol of *Listeria monocytogenes* and *Escherichia coli* O157:H7 in meat by using phages immobilized on modified cellulose membranes. *Appl. Environ. Microbiol.* 77 6379–6387 10.1128/AEM.05493-11 21803890 PMC3187159

[B2] Arachchi G. J. Cruz C. D. Dias-Wanigasekera B. M. Mcintyre L. Billington C. Hudson A. (2013). Host range and in vitro lysis of *Listeria monocytogenes* seafood isolates by bacteriophages. *Food Sci. Technol. Int.* 10.1177/1082013213497211 [Epub ahead of print] 23908393

[B3] Audurier A. Chatelain R. Chalons F. Piechaud M. (1979). Bacteriophage typing of 823 “*Listeria monocytogenes*” strains isolated in France from 1958 to 1978 (author’s transl). *Ann. Microbiol. (Paris)* 130B 179–189 119457

[B4] Audurier A. Taylor A. G. Carbonnelle B. Mclauchlin J. (1984). A phage typing system for *Listeria monocytogenes* and its use in epidemiological studies. *Clin. Invest. Med.* 7 229–232 6442645

[B5] Bakulov I. A. Kotliarov V. M. Kol’pikova T. I. (1984). Sensitivity of the phage titer increase test in detecting *Listeria*. *Zh. Mikrobiol. Epidemiol. Immunobiol.* 40–43 6506939

[B6] Bertsch D. Rau J. Eugster M. R. Haug M. C. Lawson P. A. Lacroix C. (2013). *Listeria fleischmannii* sp. nov., isolated from cheese. *Int. J. Syst. Evol. Microbiol.* 63 526–532 10.1099/ijs.0.036947-0 22523164

[B7] Bron P. A. Monk I. R. Corr S. C. Hill C. Gahan C. G. (2006). Novel luciferase reporter system for in vitro and organ-specific monitoring of differential gene expression in *Listeria monocytogenes*. *Appl. Environ. Microbiol.* 72 2876–2884 10.1128/AEM.72.4.2876-2884.2006 16597994 PMC1449049

[B8] Bigot B. Lee W. J. Mcintyre L. Wilson T. Hudson J. A. Billington C. (2011). Control of *Listeria monocytogenes* growth in a ready-to-eat poultry product using a bacteriophage. *Food Microbiol.* 28 1448–1452 10.1016/j.fm.2011.07.001 21925027

[B9] Carlton R. M. Noordman W. H. Biswas B. De Meester E. D. Loessner M. J. (2005). Bacteriophage P100 for control of *Listeria monocytogenes* in foods: genome sequence, bioinformatic analyses, oral toxicity study, and application. *Regul. Toxicol. Pharmacol.* 43 301–312 10.1016/j.yrtph.2005.08.005 16188359

[B10] Chibeu A. Agius L. Gao A. Sabour P. M. Kropinski A. M. Balamurugan S. (2013). Efficacy of bacteriophage LISTEXP100 combined with chemical antimicrobials in reducing *Listeria monocytogenes* in cooked turkey and roast beef. *Int. J. Food Microbiol.* 167 208–214 10.1016/j.ijfoodmicro.2013.08.018 24125778

[B11] Dell’Era S. Buchrieser C. Couve E. Schnell B. Briers Y. Schuppler M. (2009). *Listeria monocytogenes* L-forms respond to cell wall deficiency by modifying gene expression and the mode of division. *Mol. Microbiol.* 73 306–322 10.1111/j.1365-2958.2009.06774.x 19555455

[B12] Dorscht J. Klumpp J. Bielmann R. Schmelcher M. Born Y. Zimmer M. (2009). Comparative genome analysis of *Listeria* bacteriophages reveals extensive mosaicism, programmed translational frameshifting, and a novel prophage insertion site. *J. Bacteriol.* 191 7206–7215 10.1128/JB.01041-09 19783628 PMC2786548

[B13] Estela L. A. Sofos J. N. (1993). Comparison of conventional and reversed phage typing procedures for identification of *Listeria* spp. *Appl. Environ. Microbiol.* 59 617–619 8434928 10.1128/aem.59.2.617-619.1993 PMC202155

[B14] Fischetti V. A. (2010). Bacteriophage endolysins: a novel anti-infective to control Gram-positive pathogens. *Int. J. Med. Microbiol.* 300 357–362 10.1016/j.ijmm.2010.04.002 20452280 PMC3666336

[B15] Fleming D. W. Cochi S. L. MacDonald K. L. Brondrum J. Hayes P. S. Plikaytis B. D. (1985). Pasteurized milk as a vehicle of infection in an outbreak of listeriosis. *N. Engl. J. Med.* 14 404–407 10.1056/NEJM198502143120704 3918263

[B16] Freeman V. J. (1951). Studies on the virulence of bacteriophage-infected strains of *Corynebacterium diphtheriae*. *J. Bacteriol.* 61 675–688 14850426 10.1128/jb.61.6.675-688.1951 PMC386063

[B17] Freitag N. (2000). “Genetic tools for use with *Listeria monocytogenes*,” in *Gram-Positive Pathogens* ed. Fischetti V. A. (Washington, DC: ASM Press) 488–498

[B18] Gandhi M. Chikindas M. L. (2007). *Listeria*: a foodborne pathogen that knows how to survive. *Int. J. Food Microbiol.* 113 1–15 10.1016/j.ijfoodmicro.2006.07.008 17010463

[B19] Gerner-Smidt P. Rosdahl V. T. Frederiksen W. (1993). A new Danish *Listeria monocytogenes* phage typing system. *APMIS* 101 160–167 10.1111/j.1699-0463.1993.tb00096.x 8489767

[B20] Glaser P. Frangeul L. Buchrieser C. Rusniok C. Amend A. Baquero F. (2001). Comparative genomics of *Listeria* species. *Science* 294 849–852 10.1126/science.1063447 11679669

[B21] Graves L. M. Helsel L. O. Steigerwalt A. G. Morey R. E. Daneshvar M. I. Roof S. E. (2010). *Listeria marthii* sp. nov., isolated from the natural environment, Finger Lakes National Forest. *Int. J. Syst. Evol. Microbiol.* 60 1280–1288 10.1099/ijs.0.014118-0ijs.0.014118-0 19667380

[B22] Grundling A. Burrack L. S. Bouwer H. G. Higgins D. E. (2004). *Listeria monocytogenes* regulates flagellar motility gene expression through MogR, a transcriptional repressor required for virulence. *Proc. Natl. Acad. Sci. U.S.A.* 101 12318–12323 10.1073/pnas.0404924101 15302931 PMC514476

[B23] Guenther S. Huwyler D. Richard S. Loessner M. J. (2009). Virulent bacteriophage for efficient biocontrol of *Listeria monocytogenes* in ready-to-eat foods. *Appl. Environ. Microbiol.* 75 93–100 10.1128/AEM.01711-08 19011076 PMC2612219

[B24] Guenther S. Loessner M. J. (2011). Bacteriophage biocontrol of *Listeria monocytogenes* on soft ripened white mold and red-smear cheeses. *Bacteriophage* 1 94–100 10.4161/bact.1.2.15662 22334865 PMC3278646

[B25] Habann M. Leiman P. G. Vandersteegen K. Van den Bossche A. Lavigne R. Shneider M. M. (2014). *Listeria* phage A511, a model for the contractile tail machineries of SPO1-related bacteriophages. *Mol. Microbiol.* 92 84–99 10.1111/mmi.12539 24673724

[B26] Hagens S. De Wouters T. Vollenweider P. Loessner M. J. (2011). Reporter bacteriophage A511::celB transduces a hyperthermostable glycosidase from *Pyrococcus furiosus* for rapid and simple detection of viable *Listeria* cells. *Bacteriophage* 1 143–151 10.4161/bact.1.3.16710 22164348 PMC3225779

[B27] Hagens S. Loessner M. J. (2010). Bacteriophage for biocontrol of foodborne pathogens: calculations and considerations. *Curr. Pharm. Biotechnol.* 11 58–68 10.2174/138920110790725429 20214608

[B28] Hodgson D. A. (2000). Generalized transduction of serotype 1/2 and serotype 4b strains of *Listeria monocytogenes*. *Mol. Microbiol.* 35 312–323 10.1046/j.1365-2958.2000.01643.x 10652092

[B29] Junillon T. Vimont A. Mosticone D. Mallen B. Baril F. Rozand C. (2012). Simplified detection of food-borne pathogens: an in situ high affinity capture and staining concept. *J. Microbiol. Methods* 91 501–505 10.1016/j.mimet.2012.09.015 23017294

[B30] Kim J. W. Siletzky R. M. Kathariou S. (2008). Host ranges of *Listeria*-specific bacteriophages from the turkey processing plant environment in the United States. *Appl. Environ. Microbiol.* 74 6623–6630 10.1128/AEM.01282-08 18791016 PMC2576701

[B31] Klumpp J. Dorscht J. Lurz R. Bielmann R. Wieland M. Zimmer M. (2008). The terminally redundant, non-permuted genome of *Listeria* bacteriophage A511: a model for the SPO1-like myoviruses of gram-positive bacteria. *J. Bacteriol.* 190 5753–5765 10.1128/JB.00461-08 18567664 PMC2519532

[B32] Kretzer J. W. Lehmann R. Schmelcher M. Banz M. Kim K. P. Korn C. (2007). Use of high-affinity cell wall-binding domains of bacteriophage endolysins for immobilization and separation of bacterial cells. *Appl. Environ. Microbiol.* 73 1992–2000 10.1128/AEM.02402-06 17277212 PMC1828835

[B33] Lauer P. Chow M. Y. Loessner M. J. Portnoy D. A. Calendar R. (2002). Construction, characterization, and use of two *Listeria monocytogenes* site-specific phage integration vectors. *J. Bacteriol.* 184 4177–4186 10.1128/JB.184.15.4177-4186.2002 12107135 PMC135211

[B34] Leclercq A. Clermont D. Bizet C. Grimont P. A. Le Fleche-Mateos A. Roche S. M. (2010). *Listeria rocourtiae* sp. nov. *Int. J. Syst. Evol. Microbiol.* 60 2210–2214 10.1099/ijs.0.017376-0ijs.0.017376-0 19915117

[B35] Lenz L. L. Mohammadi S. Geissler A. Portnoy D. A. (2003). SecA2-dependent secretion of autolytic enzymes promotes *Listeria monocytogenes* pathogenesis. *Proc. Natl. Acad. Sci. U.S.A.* 100 12432–12437 10.1073/pnas.2133653100 14527997 PMC218775

[B36] Lenz L. L. Portnoy D. A. (2002). Identification of a second *Listeria* secA gene associated with protein secretion and the rough phenotype. *Mol. Microbiol.* 45 1043–1056 10.1046/j.1365-2958.2002.03072.x 12180923

[B37] Leverentz B. Conway W. S. Camp M. J. Janisiewicz W. J. Abuladze T. Yang M. (2003). Biocontrol of *Listeria monocytogenes* on fresh-cut produce by treatment with lytic bacteriophages and a bacteriocin. *Appl. Environ. Microbiol.* 69 4519–4526 10.1128/AEM.69.8.4519-4526.2003 12902237 PMC169090

[B38] Leverentz B. Conway W. S. Janisiewicz W. Camp M. J. (2004). Optimizing concentration and timing of a phage spray application to reduce *Listeria monocytogenes* on honeydew melon tissue. *J. Food Prot.* 67 1682–1686 15330534 10.4315/0362-028x-67.8.1682

[B39] Loessner M. J. (1991). Improved procedure for bacteriophage typing of *Listeria* strains and evaluation of new phages. *Appl. Environ. Microbiol.* 57 882–884 2039238 10.1128/aem.57.3.882-884.1991 PMC182812

[B40] Loessner M. J. Busse M. (1990). Bacteriophage typing of *Listeria* species. *Appl. Environ. Microbiol.* 56 1912–1918 2116763 10.1128/aem.56.6.1912-1918.1990 PMC184530

[B41] Loessner M. J. Inman R. B. Lauer P. Calendar R. (2000). Complete nucleotide sequence, molecular analysis and genome structure of bacteriophage A118 of *Listeria monocytogenes*: implications for phage evolution. *Mol. Microbiol.* 35 324–340 10.1046/j.1365-2958.2000.01720.x 10652093

[B42] Loessner M. J. Rees C. E. Stewart G. S. Scherer S. (1996). Construction of luciferase reporter bacteriophage A511::luxAB for rapid and sensitive detection of viable *Listeria* cells. *Appl. Environ. Microbiol.* 62 1133–1140 8919773 10.1128/aem.62.4.1133-1140.1996 PMC167878

[B43] Loessner M. J. Rudolf M. Scherer S. (1997). Evaluation of luciferase reporter bacteriophage A511::luxAB for detection of *Listeria monocytogenes* in contaminated foods. *Appl. Environ. Microbiol.* 63 2961–2965 9251182 10.1128/aem.63.8.2961-2965.1997 PMC168593

[B44] Loessner M. J. Wendlinger G. Scherer S. (1995). Heterogeneous endolysins in *Listeria monocytogenes* bacteriophages: a new class of enzymes and evidence for conserved holin genes within the siphoviral lysis cassettes. *Mol. Microbiol.* 16 1231–1241 10.1111/j.1365-2958.1995.tb02345.x 8577256

[B45] McLauchlin J. Audurier A. Taylor A. G. (1986). The evaluation of a phage-typing system for *Listeria monocytogenes* for use in epidemiological studies. *J. Med. Microbiol.* 22 357–365 10.1099/00222615-22-4-357 3098978

[B46] Oliveira M. Vinas I. Colas P. Anguera M. Usall J. Abadias M. (2014). Effectiveness of a bacteriophage in reducing *Listeria monocytogenes* on fresh-cut fruits and fruit juices. *Food Microbiol.* 38 137–142 10.1016/j.fm.2013.08.018 24290636

[B47] Pedulla M. L. Ford M. E. Houtz J. M. Karthikeyan T. Wadsworth C. Lewis J. A. (2003). Origins of highly mosaic mycobacteriophage genomes. *Cell* 113 171–182 10.1016/S0092-8674(03)00233-2 12705866

[B48] Rabinovich L. Sigal N. Borovok I. Nir-Paz R. Herskovits A. A. (2012). Prophage excision activates *Listeria* competence genes that promote phagosomal escape and virulence. *Cell* 150 792–802 10.1016/j.cell.2012.06.036 22901809

[B49] Rocourt J. Audurier A. Courtieu A. L. Durst J. Ortel S. Schrettenbrunner A. (1985). A multi-centre study on the phage typing of *Listeria monocytogenes*. *Zentralbl. Bakteriol. Mikrobiol. Hyg. A* 259 489–497 3931391 10.1016/s0176-6724(85)80081-x

[B50] Schmelcher M. Donovan D. M. Loessner M. J. (2012). Bacteriophage endolysins as novel antimicrobials. *Future Microbiol.* 7 1147–1171 10.2217/fmb.12.97 23030422 PMC3563964

[B51] Schmelcher M. Shabarova T. Eugster M. R. Eichenseher F. Tchang V. S. Banz M. (2010). Rapid multiplex detection and differentiation of *Listeria* cells by use of fluorescent phage endolysin cell wall binding domains. *Appl. Environ. Microbiol.* 76 5745–5756 10.1128/AEM.00801-10 20622130 PMC2935047

[B52] Schmuki M. M. Erne D. Loessner M. J. Klumpp J. (2012). Bacteriophage P70: unique morphology and unrelatedness to other *Listeria* bacteriophages. *J. Virol.* 86 13099–13102 10.1128/JVI.02350-12JVI.02350-12 22993158 PMC3497695

[B53] Smith H. W. Green P. Parsell Z. (1983). Vero cell toxins in *Escherichia coli* and related bacteria: transfer by phage and conjugation and toxic action in laboratory animals, chickens and pigs. *J. Gen. Microbiol.* 129 3121–3137 6418852 10.1099/00221287-129-10-3121

[B54] Soni K. A. Desai M. Oladunjoye A. Skrobot F. Nannapaneni R. (2012). Reduction of *Listeria monocytogenes* in queso fresco cheese by a combination of listericidal and listeriostatic GRAS antimicrobials. *Int. J. Food Microbiol.* 155 82–88 10.1016/j.ijfoodmicro.2012.01.010 22305889

[B55] Soni K. A. Nannapaneni R. (2010a). Bacteriophage significantly reduces *Listeria monocytogenes* on raw salmon fillet tissue. *J. Food Prot.* 73 32–38 20051201 10.4315/0362-028x-73.1.32

[B56] Soni K. A. Nannapaneni R. (2010b). Removal of *Listeria monocytogenes* biofilms with bacteriophage P100. *J. Food Prot.* 73 1519–1524 20819365 10.4315/0362-028x-73.8.1519

[B57] Soni K. A. Nannapaneni R. Hagens S. (2010). Reduction of *Listeria monocytogenes* on the surface of fresh channel catfish fillets by bacteriophage Listex P100. *Foodborne Pathog. Dis.* 7 427–434 10.1089/fpd.2009.0432 19958102

[B58] Tolba M. Ahmed M. U. Tlili C. Eichenseher F. Loessner M. J. Zourob M. (2012). A bacteriophage endolysin-based electrochemical impedance biosensor for the rapid detection of *Listeria* cells. *Analyst* 137 5749–5756 10.1039/c2an35988j 23085745

[B59] van der Mee-Marquet N. Loessner M. Audurier A. (1997). Evaluation of seven experimental phages for inclusion in the international phage set for the epidemiological typing of *Listeria monocytogenes*. *Appl. Environ. Microbiol.* 63 3374–3377 9292987 10.1128/aem.63.9.3374-3377.1997 PMC168643

[B60] Vázquez-Boland J. A. Kuhn M. Berche P. Chakraborty T. Dominguez-Bernal G. Goebel W. (2001). *Listeria* pathogenesis and molecular virulence determinants. *Clin. Microbiol. Rev.* 14 584–640 10.1128/CMR.14.3.584-640.2001 11432815 PMC88991

[B61] Verghese B. Lok M. Wen J. Alessandria V. Chen Y. Kathariou S. (2011). ComK prophage junction fragments as markers for *Listeria monocytogenes* genotypes unique to individual meat and poultry processing plants and a model for rapid niche-specific adaptation, biofilm formation, and persistence. *Appl. Environ. Microbiol.* 77 3279–3292 10.1128/AEM.00546-11 21441318 PMC3126449

[B62] Walcher G. Stessl B. Wagner M. Eichenseher F. Loessner M. J. Hein I. (2010). Evaluation of paramagnetic beads coated with recombinant *Listeria* phage endolysin-derived cell-wall-binding domain proteins for separation of *Listeria monocytogenes* from raw milk in combination with culture-based and real-time polymerase chain reaction-based quantification. *Foodborne Pathog. Dis.* 7 1019–1024 10.1089/fpd.2009.0475 20500083

[B63] Waldor M. K. Mekalanos J. J. (1996). Lysogenic conversion by a filamentous phage encoding cholera toxin. *Science* 272 1910–1914 10.1126/science.272.5270.1910 8658163

[B64] Wendlinger G. Loessner M. J. Scherer S. (1996). Bacteriophage receptors on *Listeria monocytogenes* cells are the N-acetylglucosamine and rhamnose substituents of teichoic acids or the peptidoglycan itself. *Microbiology* 142(Pt 4) 985–992 10.1099/00221287-142-4-985 8936325

[B65] Wommack K. E. Colwell R. R. (2000). Virioplankton: viruses in aquatic ecosystems. *Microbiol. Mol. Biol. Rev.* 64 69–114 10.1128/MMBR.64.1.69-114.2000 10704475 PMC98987

[B66] Zimmer M. Sattelberger E. Inman R. Scherer S. Calendar R. Loessner M. J. (2003). Genome and proteome of *Listeria monocytogenes* bacteriophage PSA: an unusual case for programmed +1 translational frameshifting in structural protein synthesis. *Mol. Microbiol.* 50 303–317 10.1046/j.1365-2958.2003.03684.x 14507382

[B67] Zink R. Loessner M. J. (1992). Classification of virulent and temperate bacteriophages of *Listeria* spp. on the basis of morphology and protein analysis. *Appl. Environ. Microbiol.* 58 296–302 1539980 10.1128/aem.58.1.296-302.1992 PMC195207

[B68] Zink R. Loessner M. J. Glas I. Scherer S. (1994). Supplementary *Listeria*-typing with defective *Listeria* phage particles (monocins). *Lett. Appl. Microbiol.* 19 99–101 10.1111/j.1472-765X.1994.tb00915.x 7765224

[B69] Zink R. Loessner M. J. Scherer S. (1995). Characterization of cryptic prophages (monocins) in *Listeria* and sequence analysis of a holin/endolysin gene. *Microbiology* 141(Pt 10) 2577–2584 10.1099/13500872-141-10-2577 7582018

